# Predictors of neutralizing antibody response to BNT162b2 vaccination in allogeneic hematopoietic stem cell transplant recipients

**DOI:** 10.1186/s13045-021-01190-3

**Published:** 2021-10-24

**Authors:** Lorenzo Canti, Stéphanie Humblet-Baron, Isabelle Desombere, Julika Neumann, Pieter Pannus, Leo Heyndrickx, Aurélie Henry, Sophie Servais, Evelyne Willems, Grégory Ehx, Stanislas Goriely, Laurence Seidel, Johan Michiels, Betty Willems, Adrian Liston, Kevin K. Ariën, Yves Beguin, Maria E. Goossens, Arnaud Marchant, Frédéric Baron

**Affiliations:** 1 Laboratory of Hematology, GIGA-I3, University of Liege and CHU of Liège, Liege, Belgium; 2 Department of Microbiology, Immunology and Transplantation, Laboratory of Adaptive Immunology, KU Leuven, Leuven, Belgium; 3 SD Infectious Diseases in Humans, Sciensano, 642 Engelandstraat, 1180 Ukkel, Belgium; 4 Virology Unit, Department of Biomedical Sciences, Institute of Tropical Medicine, 155 Nationalestraat, 2000 Antwerp, Belgium; 5 Division of Hematology, Department of Medicine, CHU of Liège, Liège, Belgium; 6 Institute for Medical Immunology and ULB Center for Research in Immunology (U-CRI), Université Libre de Bruxelles (ULB), Gosselies, Belgium; 7 Department of Biostatistics, University Hospital of Liège, Liège, Belgium; 8 Laboratory of Lymphocyte Signalling and Development, The Babraham Institute, Cambridge, UK; 9 Department of Hematology, University of Liège, CHU Sart-Tilman, 4000 Liège, Belgium

**Keywords:** Vaccine, BNT162b2 mRNA vaccine, SARS-CoV-2, COVID-19, Allogeneic, Hematopoietic cell transplantation, T-SNE, Switched B cells, Plasmacytoid dendritic cells

## Abstract

**Background:**

Factors affecting response to SARS-CoV-2 mRNA vaccine in allogeneic hematopoietic stem cell transplantation (allo-HCT) recipients remain to be elucidated.

**Methods:**

Forty allo-HCT recipients were included in a study of immunization with BNT162b2 mRNA vaccine at days 0 and 21. Binding antibodies (Ab) to SARS-CoV-2 receptor binding domain (RBD) were assessed at days 0, 21, 28, and 49 while neutralizing Ab against SARS-CoV-2 wild type (NT50) were assessed at days 0 and 49. Results observed in allo-HCT patients were compared to those obtained in 40 healthy adults naive of SARS-CoV-2 infection. Flow cytometry analysis of peripheral blood cells was performed before vaccination to identify potential predictors of Ab responses.

**Results:**

Three patients had detectable anti-RBD Ab before vaccination. Among the 37 SARS-CoV-2 naive patients, 20 (54%) and 32 (86%) patients had detectable anti-RBD Ab 21 days and 49 days postvaccination. Comparing anti-RBD Ab levels in allo-HCT recipients and healthy adults, we observed significantly lower anti-RBD Ab levels in allo-HCT recipients at days 21, 28 and 49. Further, 49% of allo-HCT patients versus 88% of healthy adults had detectable NT50 Ab at day 49 while allo-HCT recipients had significantly lower NT50 Ab titers than healthy adults (*P* = 0.0004). Ongoing moderate/severe chronic GVHD (*P* < 0.01) as well as rituximab administration in the year prior to vaccination (*P* < 0.05) correlated with low anti-RBD and NT50 Ab titers at 49 days after the first vaccination in multivariate analyses. Compared to healthy adults, allo-HCT patients without chronic GVHD or rituximab therapy had comparable anti-RBD Ab levels and NT50 Ab titers at day 49. Flow cytometry analyses before vaccination indicated that Ab responses in allo-HCT patients were strongly correlated with the number of memory B cells and of naive CD4^+^ T cells (*r* > 0.5, *P* < 0.01) and more weakly with the number of follicular helper T cells (*r* = 0.4, *P* = 0.01).

**Conclusions:**

Chronic GVHD and rituximab administration in allo-HCT recipients are associated with reduced Ab responses to BNT162b2 vaccination. Immunological markers could help identify allo-HCT patients at risk of poor Ab response to mRNA vaccination.

***Trial registration*:**

The study was registered at clinicaltrialsregister.eu on 11 March 2021 (EudractCT # 2021-000673-83).

**Supplementary Information:**

The online version contains supplementary material available at 10.1186/s13045-021-01190-3.

## Background

Allogeneic hematopoietic stem cell transplantation (allo-HCT) has remained the best treatment option for many patients with life-threatening hematological disorders such as acute myeloid leukemia [1]. Unfortunately, the procedure induces severe immunosuppression persisting several months to several years after transplantation, particularly in patients suffering from chronic graft-versus-host disease (GVHD). This is due to defects in B-cell, T-cell, monocyte and dendritic cell compartments [2–4]. As a consequence, infection of allo-HCT recipients with severe acute respiratory syndrome coronavirus 2 (SARS-CoV-2) causes severe forms of coronavirus disease 2019 (COVID-19) more frequently than in healthy individuals [5]. Indeed, preliminary reports showed that mortality of allo-HCT patients diagnosed with COVID-19 range from 30 to 35% 30 days after diagnosis [5, 6]. Further, allo-HCT patients can experience prolonged COVID-19 infection due to their inability to clear the virus [7]. Thus, protecting allo-HCT recipients with effective vaccination against SARS-CoV-2 is critical.

LNP-formulated messenger (m)RNA vaccine technology allows the delivery of precise genetic information together with an adjuvant effect to antigen-presenting cells [8]. Studies in mouse models have demonstrated that, after subcutaneous injection, LNP-formulated mRNA vaccines generate high levels of polyclonal antigen-specific CD4^+^ T follicular helper (TFH) cells and polyclonal antigen-specific germinal center B cells [8]. This was associated with the sustained presence of high-affinity neutralizing antibodies (Ab). Clinical studies demonstrated that Pfizer/BioNTech BNT162b2 vaccine provided 95% protection in healthy adults after injection of two doses of vaccine given three weeks apart [9]. Accordingly, this vaccine schedule induced the generation of anti-SARS-CoV-2 spike Ab in most healthy recipients [10, 11]. However, in a large cohort of kidney transplant recipients, the majority of participants failed to mount appreciable Ab responses to the Spike protein of SARS-CoV-2 following the first (85%) or the second (46%) dose of the vaccine [12, 13]. Similarly, low response to mRNA vaccination has been observed in patients with chronic lymphocytic leukemia, particularly in those given anti-CD20 antibodies in the last 12 months before vaccination (no response was observed in this subgroup of patients) [14]. These observations suggest that an important proportion of allo-HCT recipients might not mount a protective anti-spike Ab response after BNT162b2 mRNA vaccination. This prompted us to perform a phase IV study assessing the immunogenicity and safety of BNT162b2 mRNA vaccination in allo-HCT recipients. The Ab response detected in this high-risk population was compared to that of a healthy adult control population.

## Methods

### Study design

This study is a phase IV trial assessing the immunogenicity and the safety of two intramuscular injections of 30 μg BNT162b2 mRNA vaccine at day 0 and day 21 in allo-HCT recipients. Inclusion criteria included allo-HCT 3 months to 5 years prior to inclusion (any donor type), age ≥ 18 years at inclusion, and written informed consent. Exclusion criteria included HIV seropositivity, pregnancy, active malignant disease, ongoing grade III–IV acute GVHD, in vitro T-cell depletion of the graft if vaccination within 6 months after allo-HCT, rituximab administration in the 6 months prior to study inclusion, and prior documented SARS-CoV-2 infection. As per protocol, immunogenicity of the vaccine in allo-HCT recipients was compared to that in a group of 40 healthy staff members (healthy adult controls, 11 males and 29 females) included in the PICOV (Prior Infection with SARS-CoV-2) prospective cohort aimed at comparing immune response to SARS-CoV-2 mRNA vaccination in naive and previously infected residents and staff members of nursing homes in Belgium (EU Clinical Trials Register (EUdraCT 2021-000673-83)) [15, 16]. Their median age was 48 years (range 23–64 years).

### Adverse events

Adverse events were collected at each patient follow-up visit (day 1, day 21, day 28 and day 49). The following items were systematically collected: pain, redness or swelling at injection site, fever, fatigue, headache, chills, vomiting, diarrhea, muscle pain, joint pain, and use of antipyretic medication. Serious adverse events were collected and were graded according to Common Terminology Criteria for Adverse Events (CTC) version 5.0.

### Flow cytometry

After cell counting on pocH-100i automated whole blood counter (Sysmex, Kobe, Japan), PBMCs were isolated using density gradient centrifugation on Ficoll-Paque™ Plus separation medium (GE Healthcare, Illinois, USA). Subsequently, about 4 × 10^6^ PBMCs were stained for panel 1 (assessing total T cells, B cells and myeloid cells; Additional file 1: Table 1) staining whereas the remaining cells were processed for T-cell purification with EasySep™ Human T cell negative isolation kit (StemCell Technologies, Vancouver, Canada) according to the manufacturer instructions and about 3 × 10^6^ T cells were stained for panel 2 (assessing T-cell subsets, Additional file 1: Table 1) staining.

Staining of fresh PBMCs and purified T cells was carried out by incubating cells with (1) fluorochrome-conjugated Ab against surface markers, (2) live/dead cell marker and (3) fluorochrome-conjugated antibodies against intracellular markers following a fixation/permeabilization step. For surface antigen and intracellular staining, PBMCs or T cells were resuspended in 100 µl PBS 5% FBS in polystyrene 5-ml round-bottom tubes (Corning, New York, USA) and incubated with panel 1 or panel 2 antibody mixes and BD Horizon™ Brilliant Stain Buffer for 30 min at 4 °C. For live/dead cell staining, cells were resuspended in 500 µl pure PBS and incubated with 1 µl fixable viability dye and then submitted to overnight (O/N) fixation/permeabilization step with Intracellular Fixation & Permeabilization Buffer Set (eBioscience™, California, USA). Samples were analyzed the following day using a FACS LSRFortessa™ (BD Biosciences) and the BD FACSDiva™ software (BD Biosciences). The results were processed with FlowJo-V10.7.1 (FlowJo LLC, Oregon, USA). Gating strategy and cell subtype definitions are detailed in Additional file 1: Figure 1. Absolute lymphocyte counts were quantified with a pocH-100i counter. For panel 1, absolute lymphoid cell subsets (B, T and NK cells) were calculated by multiplying the absolute lymphocyte counts determined by ABX Micros 60 automated cell counter by the percentage of parental (lymphocyte gate on FSC/SSC) live cells. For panel 2, absolute counts were calculated by multiplying the absolute T-cell counts obtained in panel 1 by percentage of parental (lymphocyte gate on FSC/SSC) live cells.

### SARS-CoV-2-specific binding antibodies

SARS-Cov-2-specific binding antibodies were quantified using the FDA-approved WANTAI (Beijing Wantai Biological Pharmacy Enterprise, Beijing, China) SARS-Cov-2 Ab ELISA as indicated in the manufacturer brochure. The antigen recognized in this assay is the receptor-binding domain of SARS-CoV-2 spike protein. The limit of quantification (LOQ) of the assay is 5 IU/mL. Values below LOQ were attributed an arbitrary value of 2.5 IU/mL in the graphs and statistical analyses.

### SARS-CoV-2 neutralizing antibodies

SARS-CoV-2 neutralizing antibodies were quantified as previously reported [17]. Briefly, serial dilutions of heat-inactivated serum (1/50-1/25600 in EMEM supplemented with 2 mM L-glutamine, 100 U/ml-100 μg/ml of Penicillin–Streptomycin and 2% fetal bovine serum) were incubated during 1 h (37 °C, 7% CO_2_) with 3xTCID100 of (i) a wild type (WT) Wuhan strain (2019-nCoV-Italy-INMI1, reference 008 V-03893). Sample-virus mixtures and virus/cell controls were added to Vero cells (18.000 cells/well) in a 96-well plate and incubated for five days (37 °C, 7% CO_2_). The cytopathic effect caused by viral growth was scored microscopically. The Reed–Muench method was used to calculate the neutralizing Ab titer that reduced the number of infected wells by 50% (NT50), which was used as a proxy for the neutralizing Ab concentration in the sample. Values below LOQ were arbitrarily attributed a value of 25 in the graphs and statistical analyses.

### Data analyses

#### Unsupervised flow cytometry analyses

The concatenated data set was analyzed through successive FlowSOM [18] clustering and t-SNE representation after exporting similar event numbers for each sample per condition group as previously reported [19, 20]. For B-cell subset analyses two samples containing few B cells (144 and 502 cells instead of 2700 cells in all other samples) were nevertheless included in the t-SNE analysis to avoid creating informative censuring. For panel 1, lineage markers (CD3, CD14, CD16, and CD19) were first used to separate leukocyte subsets. Secondly, additional markers (CD27, IgD, CD11c, CD86, and HLA-DR for B-cell subset analyses and HLA-DR, Siglec-F, CD16, CD86, CD141, CD14, CD11c, and CD123 for myeloid cell subsets) were used to distinguish phenotypic clusters of each leukocyte subset, again using FlowSOM and t-SNE. Three samples (1 nonresponder and 2 responders) were excluded for total leukocyte t-SNE analysis and five samples (2 nonresponders and 3 responders) were excluded for myeloid cell t-SNE analysis due to staining artefacts. The characteristics of each identified cluster were assessed by means of histograms and heatmaps. Comparisons between groups (responders (defined as anti-RBD Ab > 5 IU/mL) versus nonresponders at day 21) were performed with tests on the cross‐entropy distributions of the t-SNE representations of each group. In brief, for the original and t‐SNE space of each t-SNE plot, a probability per data point was calculated following the same approach as in the t-SNE algorithm. From these point probabilities, the distribution of cross‐entropy in the t-SNE space relative to the original space was obtained for each group represented in the plot. All pairwise comparisons between groups were evaluated with Kolmogorov–Smirnov tests on the difference between the cross‐entropy distributions. Dendrograms were obtained from hierarchical clustering, using as distance the Kolmogorov–Smirnov statistic, that is, the L‐infinity distance between the cross‐entropy distributions.

### Statistical analyses

Comparisons of Ab titers between various groups were done with the Mann–Whitney test. Comparisons of frequencies (%) of FlowSOM clusters between various groups were also done with the Mann–Whitney test. Correlations between age, time from HCT to vaccination, absolute immune cell counts and Ab titers were done with the Spearman r test. Comparison of the proportion of responding patients according to chronic GVHD was done with the Fisher’s exact test. Variances between samples were calculated using the F test. Analyses of clinical factors (i.e., the presence or not of moderate/severe chronic GVHD, delay from allo-HCT to vaccination, patient age and rituximab administration within 1 year before vaccination) associated with Ab response and Ab titers were performed using univariate and multivariate logistic regression (with Firth correction when indicated) and univariate and multivariate linear regression, respectively. For these analyses, delay from HCT to vaccination and Ab titers underwent logarithmic transformation. Multivariate linear regressions were also used to assess the associations between baseline counts of class-switched memory B cells, naive CD4+T cells and TFH cells and Ab levels at day 49. *P* values < 0.05 were considered as statistically significant and all *P* values were 2-sided. Statistical analyses were carried out with Graphpad Prism 9.0 (Graphpad Software, San Diego, CA, USA) and SAS version 9.4.

## Results

### Patients

We report here the data of the first 40 patients included in the study. Their characteristics are described in Table 1. Briefly, median age at vaccination was 60 years (range 26–76 years). Median time from allo-HCT to vaccination was 31 months (range 5–51 months). At the time of the first vaccination, 14 patients were still on systemic immunosuppressive treatment either as GVHD prevention (*n* = 5) or as treatment of moderate/severe chronic GVHD (*n* = 9). Seven patients were given rituximab in the year before the first vaccination, including 1 of the 9 patients with ongoing moderate/severe chronic GVHD and 6 patients without ongoing moderate/severe chronic GVHD. All but patient #25 received the 2 doses of the vaccine at a 3-week interval as scheduled. Patient #25 was diagnosed with COVID-19 on day 6 after the first vaccination and did not receive the second dose of the vaccine (see below). He already had detectable Ab (low titer) before vaccination, suggesting an ongoing infection. Two other patients had detectable anti-RBD Ab before vaccination (moderate titers), most likely reflecting prior SARS-CoV-2 infection.

**Table 1:** Characteristics of the patients (*n* = 40)

Age at vaccination (years); median (min, p25, p75, max)	60 (26, 54, 69, 76)
Sex (# males/# females)	19/21
Delay between vaccination and transplantation (months); median (min, p25, p75, max)	31 (6, 14, 42, 57)
Donor type (# MSD/MUD/ MMUD/Haplo)	8/26/1/5
Donor age at transplantation (years); median (min, p25, p75, max)	34 (18, 23, 46, 62)
Conditioning regimen (# patients)	
Fludarabine + 2 Gy TBI	5
Fludarabine + Melphalan	18
Fludarabine + busulfan	4
Cyclophosphamide + 12 Gy TBI	6
Thiotepa + busulfan + fludarabine	2
Sequential	3
Fludarabine + Cyclophosphamide + 2 or 4 Gy TBI	2
ATG (# yes/no)	29/11
PTCY (# yes/no)	6 /34
Chronic GVHD	
Never/only mild	29
Prior moderate/severe solved*	2
Ongoing moderate/severe	9
Rituximab (none or ≥ 2yrs, ≥ 1 but < 2 yrs, > 6 months but < 1 yr), # of patients	28, 5, 7
Systemic immunosuppression at inclusion	
None	26
Tacrolimus	5
Photopheresis	1
Photopheresis + mPDN < 32 mg/day	1
MMF	1
MMF + mPDN < 32 mg/day	1
Sirolimus	1
Sirolimus + mPDN < 32 mg/day	2
Photopheresis + ruxolitinib	2

* and > 3 months out of systemic immunosuppression. MSD, HLA-identical sibling donor; MUD, 10/10 HLA-matched unrelated donor; MMUD, 1/10 HLA-mismatched unrelated donor; Haplo, HLA-haploidentical donor; TBI, total body irradiation; ATG, anti-thymocyte globulin; PTCY, post-transplant cyclophosphamide; MMF, mycophenolate mofetil; mPDN, methyl-prednisolone

### Adverse events

Most frequent solicited adverse events recorded during the 49 days after the first vaccination included pain at the site of vaccination (86% of the patients), fatigue (41%), headache (30%), myalgia (28%), and chills (15%) (Additional file 1: Figure 2). Paracetamol was taken by 45% of the patients. Patient #6 had an extensive rash 15 days after the first vaccination treated with a short course of steroids. Patient #18 had a noninfectious exacerbation of chronic obstructive pulmonary disease on day 44 after the first vaccination, treated with a short course of steroids. Patient #40 was diagnosed on day 21 after the first vaccination (the day of the second vaccination) with a deep venous thrombosis linked to an implantable venous access system and 2 days later with grade III hyperkalemia. No GVHD flare was observed. Three serious adverse events (SAE) were recorded during the first 49 days following injection of the first dose of the vaccine. Patients #6 was diagnosed with probable lung aspergillosis on day 7 after the first vaccination that was treated with voriconazole. Patient #22 was diagnosed with cytogenetic relapse of his AML on day 28 after the first vaccination. Patient # 39 was diagnosed with a fracture of the tibia and fibula following a fall from a stepladder. These 3 SAEs were not considered related to the vaccination.

### SARS-CoV-2-specific RBD antibodies

**Fig. 1:**
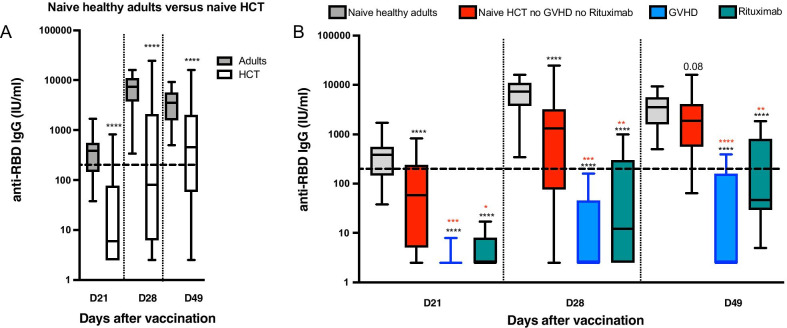
**Anti-receptor-binding domain (RBD) SARS-CoV-2 IgG titers following BNT162b2 mRNA vaccination.** All allo-HCT patients received the first vaccine on day 0 and 39 of the 40 patients received the second vaccine on day 21. **a** Comparison of Ab levels in staff members of nursing homes naive of SARS-CoV-2 infection (*n* = 40, gray bars) versus naive allo-HCT recipients (*n* = 37, white bars). **b** Comparison of Ab levels in healthy adults (*n* = 40, gray bars) versus in allo-HCT recipients naïve of COVID-19 without moderate/severe chronic GVHD and not given rituximab in the year before the first vaccination (*n* = 22, red bars), in naive allo-HCT recipients with moderate/severe chronic GVHD (*n* = 9, blue bars; please note that 8 and 5 patients had Ab levels < 5 IU/mL on days 21 and 49, respectively; this include 1 patient given rituximab within the year before the first vaccination), and in naive allo-HCT recipients without moderate/severe chronic GVHD and given rituximab within the year before the first vaccination (*n* = 6, green bars). The horizontal broken lines show the limit of possibly protective levels (200 IU/mL). *P value < 0.05; **P value < 0.01; ***P value < 0.001; ****P value < 0.0001. For **b**, black stars refer to comparisons with naive healthy adults, while red stars refer to comparisons to naive allo-HCT recipients without moderate/severe chronic GVHD and not given rituximab in the year before the first vaccination

The 3 patients with detectable anti-RBD Ab before vaccination had high (> 1000 IU/mL) Ab titers at days 21, 28 and 49 (Additional file 1: Figure 3A). At day 21, 20 of the 37 SARS-CoV-2 naive allo-HCT patients (54%) versus all 40 healthy adults had detectable anti-RBD Ab. At day 49, 32 allo-HCT recipients (86%) versus all healthy adults had detectable anti-RBD Ab. Comparing anti-RBD Ab levels in allo-HCT recipients and healthy adults, we observed significantly lower anti-RBD Ab levels in allo-HCT recipients at days 21, 28 and 49 (Fig. 1a). Further, there was a larger variance of anti-RBD Ab levels among allo-HCT patients than among healthy adults (F-test for equality of two variances yielded a *P* = 0.0005 at day 49). This prompted us to look for factors associated with Ab levels in allo-HCT recipients.

We first observed that patients with ongoing moderate/severe chronic GVHD had lower anti-RBD Ab levels than those with mild chronic GVHD or none. Specifically, 19 out of 28 patients without versus 1 out of 9 patients with ongoing moderate/severe chronic GVHD had detectable anti-RBD Ab at day 21 (*P* = 0.0055). At day 49, the figures were 28 out of 28 patients versus 4 out of 9 patients (*P* = 0.0003) (Additional file 1: Figure 3B, C). In addition, Ab titers were significantly lower in patients with than in those without moderate/severe chronic GVHD at days 21 (*P* = 0.002), 28 (*P* = 0.002) and 49 (*P* < 0.001) (Fig. 1b).

We then looked at the impact of rituximab on Ab responses in the cohort of naive allo-HCT patients without moderate/severe chronic GVHD (*n* = 28). We observed that those given rituximab < 1 year before vaccination (*n* = 6) had lower anti-RBD Ab levels than the remaining 22 patients on days 21, 28 and 49 after vaccination (*P* < 0.05 at each time point) (Fig. 1b).

We next assessed the impact of age in allo-HCT recipients on anti-RBD Ab levels. We observed a negative correlation between COVID-19 naive allo-HCT patient age (*n* = 37) and anti-RBD Ab levels at day 21 (Spearman *r* = − 0.36, *P* = 0.029), day 28 (Spearman *r* = − 0.38, *P* = 0.019) and day 49 (Spearman *r* = − 0.38, *P* = 0.020; univariate linear regression, *P* = 0.029). In addition, there was a weak correlation between time from allo-HCT to vaccination and anti-RBD Ab levels at day 21 (Spearman *r* = 0.41, *P* = 0.012), day 28 (Spearman *r* = 0.38, *P* = 0.022) and day 49 (Spearman *r* = 0.31, *P* = 0.06; univariate linear regression, *P* = 0.13).

In multivariate analysis, ongoing moderate/severe chronic GVHD (*n* = 9) was the only factor significantly associated with an absence of response to the vaccine (i.e., no detectable anti-RBD Ab at day 49; OR = 0.014, *P* = 0.018) while moderate/severe chronic GVHD (*n* = 9; Estimate = -3.87, *P* < 0.0001) and rituximab administration within the year before vaccination (*n* = 7; Estimate -2.79, *P* = 0.0004) were each associated with lower anti-RBD Ab titers (Additional file 1: Table 2).

Finally, we compared anti-RBD Ab levels in naive healthy adults and naive allo-HCT recipients without ongoing moderate/severe chronic GVHD and not given rituximab the first year before the first vaccination (*n* = 22). We observed that such allo-HCT patients had lower anti-RBD Ab levels at day 21 (*P* < 0.0001) and day 28 (*P* < 0.0001) but more comparable anti-RBD Ab levels at day 49 (*P* = 0.08) than healthy adults (Fig. 1e).

### SARS-CoV-2 neutralizing antibodies

**Fig. 2:**
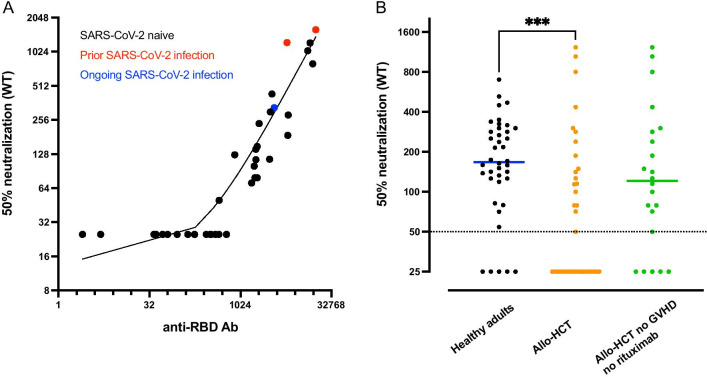
**Lower neutralizing antibody responses in allo-HCT patients than in healthy adults.** **a** Correlation between anti-RBD Ab and 50% neutralizing Ab titers of SARS-CoV-2 wild type at day 49 in allo-HCT patients (*n* = 40). The red dots show the data from the 2 patients with prior SARS-CoV-2 infection while the blue dot shows the data of the patient with ongoing SARS-CoV-2 infection at the first vaccination. **b** 50% neutralizing Ab titers of SARS-CoV-2 wild type at day 49 in SARS-CoV-2 naive healthy adults (staff members of nursing homes, *n* = 40, black dots) versus SARS-CoV-2 naive allo-HCT patients (*n* = 37, orange dots) and SARS-CoV-2 naive allo-HCT patients without moderate/severe chronic GVHD and rituximab administration in the year before vaccination (*n* = 22, green dots). The horizontal solid lines show the median and the broken line shows the lower limit of quantification (LLOQ, 1/50). *P value < 0.05; **P value < 0.01; ***P value < 0.001; ****P value < 0.0001

There was a strong correlation between neutralizing Ab titers that reduced the number of infected well by WT SARS-CoV-2 wild type by 50% (NT50) and anti-RBD Ab titers at day 49 (Spearman *r* = 0.93, *P* < 0.0001) (Fig. 2a). The 3 patients with prior (*n* = 2, they also had detectable NT50 Ab at day 0) or ongoing (*n* = 1) SARS-CoV-2 infection had detectable NT50 Ab at day 49 (Fig. 2a). Restricting the analysis to the 37 SARS-CoV-2 naive allo-HCT patients, 19 patients had undetectable NT50 Ab at day 49. This includes the 9 patients with moderate/severe chronic GVHD (*P* = 0.001 compared to patients without moderate/severe chronic GVHD). Further, only 1 out of 6 patients without chronic GVHD given rituximab < 1 year before vaccination had detectable NT50 Ab at day 49 (data not shown).

In multivariate analysis, moderate/severe chronic GVHD (*n* = 9) was associated with undetectable NT50 Ab at day 49 (OR = 0.023, *P* = 0.025) while longer time from allo-HCT to vaccination was associated with detectable NT50 Ab at day 49 (OR = 5.72, *P* = 0.048) (Additional file 1: Table 2). In addition, moderate/severe chronic GVHD (*n* = 9, Estimate = − 1.18, *P* = 0.001), rituximab administration within the year before vaccination (*n* = 7, Estimate − 0.97, *P* = 0.01) and older age (Estimate − 0.027, *P* = 0.016) were each associated with lower NT50 Ab titers, while the opposite was seen for longer time from allo-HCT to vaccination (Estimate 0.77, *P* = 0.0006) (Additional file 1: Table 2).

We next compared NT50 Ab in naive healthy adults and naive allo-HCT recipients at day 49 after vaccination. Thirty-five of the 40 adult controls (88%) versus 18 of 37 allo-HCT patients had detectable NT50 Ab at day 49 (*P* = 0.0004). Further, we observed that allo-HCT recipients had significantly lower NT50 Ab titers than healthy adults (*P* = 0.0004, Fig. 2b). Restricting the analysis to patients without moderate/severe chronic GVHD and not given rituximab in the year before to vaccination, the difference with healthy adults was no longer statistically significant (*P* = 0.25).

### Association between immune cell subsets at baseline and Ab response to vaccination

**Fig. 3:**
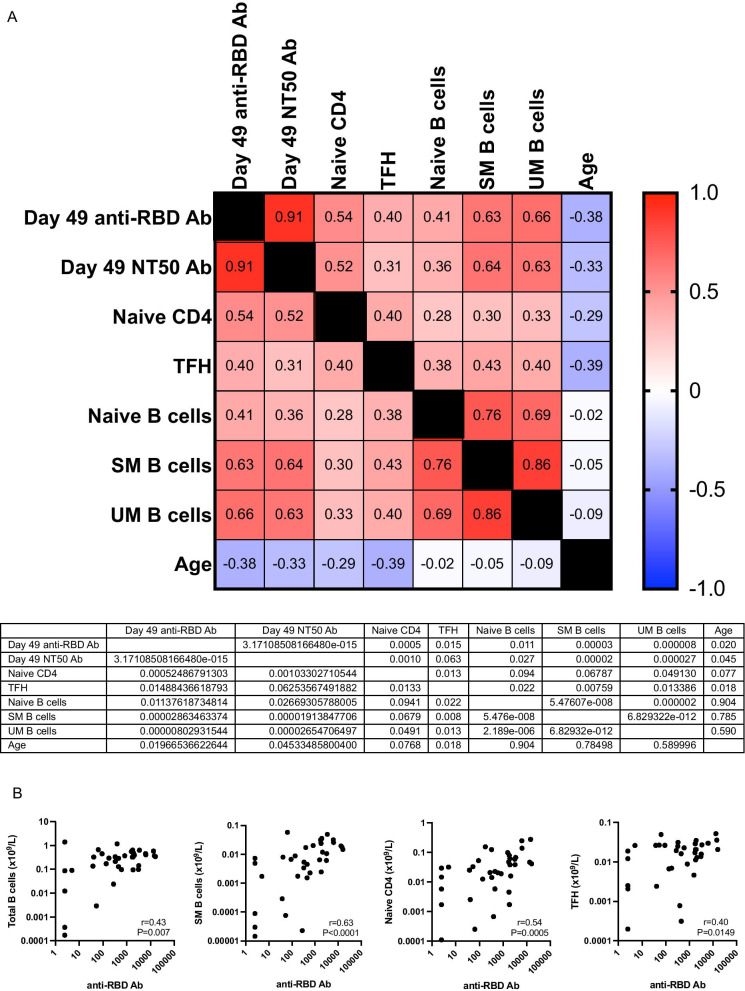
**Correlations between baseline immune cell counts and Ab response at day 49**. **a** Spearman *r* correlation matrix between day 49 anti-RBD Ab levels, day 49 50% neutralizing Ab titers of SARS-CoV-2 wild type, immune cell counts, and age in SARS-Cov2 naive allo-HCT recipients (*n* = 37). RBD, receptor-binding domain; Ab, antibody; SM B cells, class-switched memory B cells; UM B cells, class-unswitched memory B cells; TFH, T follicular helper cells. **b** Individual correlation between day 49 anti-RBD levels and total B-cell counts, class-switched memory B-cell counts, naive CD4^+^ T-cell counts and T follicular helper cell counts (Spearman *r* correlation coefficient and *p* values are shown in the graphs; *n* = 37)

We first compared baseline flow cytometry data in the 37 SARS-CoV-2 naive patients with anti-RBD Ab ≤ or > 5 IU/mL at day 21 using unsupervised flow cytometry analyses consisting of successive FlowSOM clustering and t-SNE representation. Looking first at lineage-specific markers (B, T, NK cells versus myeloid cells) we observed a trend for lower B-cell frequencies in nonresponders (*P* = 0.078, Additional file 1: Figure 4). Looking then at B-cell subsets, 5 subpopulations were identified by FlowSOM (Fig. 3a, b) including 4 subpopulations of naive B cells and a cluster of class-switched memory B cells (green dots in Fig. 3a). We observed that responders (blue dots in Fig. 3c, d and blue bars in Fig. 3e) had a higher frequency of class-switched memory B cells than nonresponders (red dots in Fig. 3c, d and red bars in Fig. 3e, median 6.7% versus 3.4%, *P* = 0.008) (Additional file 1: Figure 5). Finally, looking at myeloid cell subsets, we observed that day 21 responders had a significantly higher frequency of plasmacytoid dendritic cells among myeloid cells than nonresponders (Additional file 1: Figure 6).

The observed association between day 21 Ab response and class-switched memory B cell frequencies prompted us to examine whether absolute B cell and B cell subset counts calculated using manual gating correlated with anti-RBD Ab levels. We observed a weak correlation between absolute B cell counts and anti-RBD Ab levels: at day 21 (Spearman *r* = 0.34, *P* = 0.039), day 28 (Spearman *r* = 0.39, *P* = 0.02) and day 49 (Spearman *r* = 0.43, *P* = 0.007). A weak correlation was also observed with naive B cells: at day 21 (Spearman *r* = 0.31, *P* = 0.06), day 28 (Spearman *r* = 0.37, *P* = 0.02) and day 49 (Spearman *r* = 0.41, *P* = 0.01). There was however a much stronger correlation between class-switched memory B cell counts and anti-RBD Ab levels at day 21 (Spearman *r* = 0.56, *P* = 0.0003), day 28 (Spearman *r* = 0.53, *P* = 0.0007) and day 49 (Spearman *r* = 0.63, *P* < 0.0001). Unswitched memory B cell counts correlated also with anti-RBD Ab levels: at day 21 (Spearman *r* = 0.51, *P* = 0.0011), day 28 (Spearman *r* = 0.53, *P* = 0.0008) and day 49 (Spearman *r* = 0.66, *P* < 0.0001) (Fig. 3). Finally, looking at associations between B cell subset frequencies among absolute lymphocytes and anti-RBD Ab levels at day 49, we observed significant correlations with percentage of unswitched memory B cell (Spearman *r* = 0.62, *P* < 0.0001) and of class-switched memory B cell (Spearman *r* = 0.57, *P* = 0.0003), but not with percentage of naive B cells (Spearman *r* = 0.27, *P* = 0.1) (Additional file 1: Figure 7).

We then checked whether absolute T-cell counts correlated with anti-RBD Ab levels. We observed no correlation between absolute T-cell counts, absolute CD8^+^ T-cell counts or absolute Treg counts and Ab levels (data not shown). A weak correlation was observed between absolute CD4^+^ T-cell counts and Ab levels at day 21 (Spearman *r* = 0.27, *P* = 0.10), day 28 (Spearman *r* = 0.36, *P* = 0.03) and day 49 (Spearman *r* = 0.33, *P* = 0.048). A much stronger correlation was observed with absolute naive CD4^+^ T-cell counts: at day 21 (Spearman *r* = 0.53, *P* = 0.0008), day 28 (Spearman *r* = 0.55, *P* = 0.0004) and day 49 (Spearman *r* = 0.54, *P* = 0.0005) (Fig. 3). Interestingly, a correlation between baseline follicular helper T (TFH) cell counts and Ab levels was also observed and this correlation increased between day 21 and day 49: at day 21 (Spearman *r* = 0.25, *P* = 0.14), day 28 (Spearman *r* = 0.33, *P* = 0.046) and 49 (Spearman *r* = 0.40, *P* = 0.015) (Fig. 3). Looking at associations between T cell subset frequencies among absolute lymphocytes and anti-RBD Ab levels at day 49, we observed significant correlations with percentage of naive CD4^+^ T cells (Spearman *r* = 0.46, *P* = 0.0038) but not with percentage of TFH (Spearman *r* = 0.21, *P* = 0.2) (Additional file 1: Figure 7).

Finally, we performed multivariate linear regression analysis to assess whether baseline counts of class-switched memory B cells, naive CD4^+^ T cells and TFH cells independently correlated with Ab titers at day 49. We observed that counts of class-switched memory B cells (*P* = 0.0006) and of naive CD4^+^ T cells (*P* = 0.016) were independently associated with high anti-RBD Ab levels, while the association with TFH cells was no longer statistically significant (*P* = 0.4). Similarly, counts of class-switched memory B cells (*P* = 0.012) and of naive CD4^+^ T cells (*P* = 0.044) were independently associated with high anti-RBD NT50 titers, while the association with TFH cells was not statistically significant (*P* = 0.3).

### Postvaccination COVID-19

Two patients were diagnosed with COVID-19 after vaccination. Patient #25 was diagnosed with COVID-19 (B.1.1.7 variant) on day 6 after the first vaccination (as mentioned above, he already had some detectable anti-RBD IgG Ab the day of vaccination). He had mild disease (cough and dyspnea on exertion). His PCR was negative on day 30 but had again a slightly positive PCR on day 55 after the first vaccination. Patient #23 was diagnosed with COVID-19 (B.1.1.7 variant) on day 38 after the second vaccination. Interestingly, she had no detectable anti-RBD Ab at day 28 after the first vaccination (day 7 after the second vaccination), but had anti-RBD IgG titers of 64 IU/ml on day 49. She was pauci-symptomatic (sore throat and mild asthenia). Her viral load decreased from between 10^5^ to 10^7^ RNA copies/ml on day 42 to < 10^3^ RNA copies/ml on day 49 after the first vaccination.

## Discussion

The immunogenicity of SARS-CoV-2 mRNA vaccination in allo-HCT recipients as well as factors affecting Ab response to the vaccine in this population remains to be fully elucidated. Therefore, we performed a phase IV study of vaccination in allo-HCT recipients transplanted 3 months to 5 years before vaccination. Several observations were made.

A first observation of our study was that most allo-HCT recipients responded to the vaccine. Specifically, the response rate after one and two doses was 54% and 86%, respectively, versus 100% after one and two doses in our healthy adult control group. The response rate in our cohort of allo-HCT recipients is higher than what has been observed in kidney transplant recipients in whom antibody responses to SARS-CoV-2 mRNA vaccine was 15% after 1 dose and 54% after 2 doses [13]. This is, however, in concordance with a recent report assessing the immunogenicity of the SARS-CoV-2 mRNA vaccine in a large cohort of patients with hematological malignancies which observed high antibody response as well as high titers in most allo-HCT recipients, although the exact proportion of allo-HCT patients who responded to the vaccine was not specified in that study [21]. This is also in concordance with a recent study in allo-HCT patients in which 82% of the allo-HCT patients had detectable anti-RBD Ab 28 days after the second dose [22]. Comparing anti-RBD Ab levels in allo-HCT patients and in adult controls, we observed significantly lower Ab levels in allo-HCT patients at each time point. In addition, only half of allo-HCT patients had detectable neutralizing Ab against WT SARS-CoV-2 at day 49 while allo-HCT recipients had significantly lower neutralizing Ab titers than healthy controls at that time point. Restricting the analyses to allo-HCT patients without GVHD and without rituximab administration in the year before vaccination, we still observed significantly lower Ab levels in allo-HCT recipients at day 21 but Ab levels were comparable to healthy controls at day 49. The same was true for neutralizing Ab titers. These results emphasize the importance of a timely second vaccination in this population.

A second important observation was that ongoing moderate/severe chronic GVHD was associated with a lower Ab response to the vaccine both after 1 and 2 vaccine doses. Indeed, 5 out of 9 patients with moderate/severe chronic GVHD failed to develop anti-RBD Ab following the 2 vaccine doses, while 3 additional patients had anti-RBD Ig titers below 200 IU/mL on day 49. Accordingly, none of the patients with ongoing moderate/chronic GVHD had neutralizing antibodies against the WT SARS-CoV-2 at day 49. The impaired response to mRNA vaccine in patients with chronic GVHD is likely due to delayed/disrupted return to immune homeostasis in chronic GVHD patients leading to defects in key cell populations. Indeed, it is well known that chronic GVHD (and its treatment) has a profound impact on immunity after allo-HCT, affecting many cell subtypes such as B cells, CD4^+^ T cells, naive CD4^+^ T cells, TfH and CD8^+^ T cells [8, 23, 24]. Alternatively, ongoing chronic GVHD might distract from coordinated immune response to mRNA vaccine by driving concurrent immune responses against host antigens. Previous clinical studies have observed lower response rates to pneumococcal conjugate vaccine, hepatitis B vaccine, tetanus vaccine and influenza vaccine in patients with chronic GVHD [25–28]. However, chronic GVHD had a modest or no impact on the response to diphtheria and hemophilus influenza type B vaccination [27].

In our cohort, all allo-HCT patients without chronic GVHD had detectable Ab on day 49 after vaccination. However, only 64% of them had neutralizing antibodies against the WT SARS-CoV-2 at day 49. Looking at factors associated with Ab levels in the subgroup of naive patients without moderate/severe chronic GVHD, we observed that patients given rituximab 6 months to 1 year before vaccination had lower Ab titers. This is in line with what was observed in patients with chronic lymphocytic leukemia [14], B-cell non-Hodgkin lymphoma [29], and multiple sclerosis [30]. We also observed a negative correlation between Ab levels and age, particularly in the subgroup of patients without chronic GVHD and without recent rituximab administration. This correlation is likely to be at least partly related to the different pattern of immune reconstitution following allo-HCT in younger versus older patients, including lower recovery of naive T-cell counts due to thymus atrophy [23].

A limitation of the current study is that we did not assess SARS-CoV-2-specific T cell responses to the vaccine. In a recent study, cellular response (assessed by an ELISpot assay) was detected in only 19% of allo-HCT patients given two doses of the BNT162b2 vaccine [31]. Further studies should assess whether common or distinct parameters predict Ab and T cell responses to mRNA vaccination in all-HCT recipients.

Importantly, we observed that the baseline absolute cell counts of several cell subsets correlated to Ab response. Specifically, we observed a strong correlation with switched and unswitched memory B cell counts at baseline. As expected, these cell subtypes represented a relatively small proportion of B cells in our cohort given that (in contrast to T-cell) B-cell recovery after allo-HCT follows the ontogeny with the early rise of B cells following allo-HCT being due nearly exclusively to naive B cells [2, 3, 32]. Correlations of Ab response with absolute and naive B-cell counts were weaker. Further studies are needed to determine whether these observations are due to cross-reactivity of preexisting memory B cells to the vaccine or whether these correlations between memory B cells and Ab responses are the reflection of a better general immunity in these patients. Interestingly, two factors known for impacting B-cell recovery after allo-HCT (i.e., GVHD and rituximab administration) were also associated with lower Ab response to the vaccine in our cohort [2].

In our study, absolute counts of naive CD4^+^ T cells also strongly correlated with anti-RBD Ab levels. Such an association between naive CD4^+^ T cell response and Ab levels has previously been observed in allo-HCT recipients receiving the AS03-adjuvanted influenza A/09/H1N1 vaccine [28]. Further, this is in line with the important role of CD4^+^ T cells in the response to mRNA vaccines. Indeed, mouse models have shown that mRNA vaccines induce strong CD4^+^ T cell responses, including antigen-specific THF responses, leading to potent and long-lived Ab responses [8]. Interestingly, we also observed a correlation between absolute TFH counts and Ab response in our cohort of patients, although this correlation was weaker than the one observed with naive CD4^+^ T-cell counts. This association between response to mRNA vaccine and naive CD4^+^ T-cell counts suggests a share mechanisms for poor responses to the vaccine in aged and in allo-HCT (and particularly those with chronic GVHD) patients [33].

## Conclusions

In summary, we observed that allo-HCT patients without moderate/severe chronic GVHD and not given rituximab within 1 year before vaccination had comparable anti-RBD Ab levels to those of healthy adults following two doses of the vaccine. However, moderate/severe chronic GVHD and rituximab administration were associated with lower Ab levels in allo-HCT recipients. Administration of a third dose of the vaccine should be investigated in allo-HCT patients with low anti-RBD Ab levels or low neutralizing Ab titers. Indeed a recent study observed that 52% of allo-HCT patients with low Ab responses (defined as anti-RBD Ab levels < 4160 AU/mL) following two doses of the BNT162b2 vaccine responded to a third dose administered 51 ± 22 days after the second dose [34]. Importantly, using baseline flow cytometry analyses we observed that absolute counts of several cell subtypes including switched and unswitched memory B cells, naive CD4^+^ T cells and TFH correlated with anti-RBD Ab and responses and neutralizing Ab against WT SARS-CoV-2.

## Supplementary Information

**Additional file 1**. Additional tables (n = 2) and figures (n = 7).

## Data Availability

Clinical data, antibody response and flow cytometry data are available upon reasonable request to the corresponding author.

## Abbreviations

Ab: Antibody
allo-HCT: Allogeneic hematopoietic stem cell transplantation
COVID-19: Coronavirus disease 2019
CTC: Common Terminology Criteria for Adverse Events
DC: Dendritic cells
FBS: Fetal bovine serum
GVHD: Graft-versus-host disease
LOQ: Limit of quantification
NT50: Neutralizing Ab titer that reduced the number of infected well by SARS-CoV-2 wild type by 50%
PBMC: Peripheral blood mononuclear cells
PBS: Phosphate-buffered saline
RBD: Receptor binding domain
SARS-CoV-2: Severe acute respiratory syndrome coronavirus 2
TFH: T follicular helper cells
T-SNE: T-distributed stochastic neighbor embedding
WT: Wild type

## References

[1] Baron F, Efficace F, Cannella L, Willemze R, Vignetti M, Muus P (2020). Long-term follow-up of a trial comparing post-remission treatment with autologous or allogeneic bone marrow transplantation or intensive chemotherapy in younger acute myeloid leukemia patients. Haematologica.

[2] Bosch M, Khan FM, Storek J (2012). Immune reconstitution after hematopoietic cell transplantation. Curr Opin Hematol.

[3] Hannon M, Beguin Y, Ehx G, Servais S, Seidel L, Graux C (2015). Immune recovery after allogeneic hematopoietic stem cell transplantation following Flu-TBI versus TLI-ATG conditioning. Clin Cancer Res Off J Am Assoc Cancer Res.

[4] Peric Z, Cahu X, Malard F, Brissot E, Chevallier P, Guillaume T (2015). Peripheral Blood plasmacytoid dendritic cells at day 100 can predict outcome after allogeneic stem cell transplantation. Biol Blood Marrow Transplant J Am Soc Blood Marrow Transplant.

[5] Sharma A, Bhatt NS, St Martin A, Abid MB, Bloomquist J, Chemaly RF (2021). Clinical characteristics and outcomes of COVID-19 in haematopoietic stem-cell transplantation recipients: an observational cohort study. Lancet Haematol.

[6] Xhaard A, Xhaard C, D’Aveni M, Salvator H, Chabi M-L, Berceanu A (2021). Risk factors for a severe form of COVID-19 after allogeneic haematopoietic stem cell transplantation: a Société Francophone de Greffe de Moelle et de Thérapie cellulaire (SFGM-TC) multicentre cohort study. Br J Haematol.

[7] Roedl K, Heidenreich S, Pfefferle S, Jarczak D, Urbanowicz TT, Nörz D (2021). Viral dynamics of SARS-CoV-2 in critically ill allogeneic hematopoietic stem cell transplant recipients and immunocompetent patients with COVID-19. Am J Respir Crit Care Med.

[8] Pardi N, Hogan MJ, Naradikian MS, Parkhouse K, Cain DW, Jones L (2018). Nucleoside-modified mRNA vaccines induce potent T follicular helper and germinal center B cell responses. J Exp Med.

[9] Polack FP, Thomas SJ, Kitchin N, Absalon J, Gurtman A, Lockhart S (2020). Safety and Efficacy of the BNT162b2 mRNA Covid-19 Vaccine. N Engl J Med.

[10] Walsh EE, Frenck RWJ, Falsey AR, Kitchin N, Absalon J, Gurtman A (2020). Safety and immunogenicity of two RNA-based Covid-19 vaccine candidates. N Engl J Med.

[11] Sadarangani M, Marchant A, Kollmann TR (2021). Immunological mechanisms of vaccine-induced protection against COVID-19 in humans. Nat Rev Immunol.

[12] Boyarsky BJ, Werbel WA, Avery RK, Tobian AAR, Massie AB, Segev DL (2021). Immunogenicity of a single dose of SARS-CoV-2 messenger RNA vaccine in solid organ transplant recipients. JAMA.

[13] Boyarsky BJ, Werbel WA, Avery RK, Tobian AAR, Massie AB, Segev DL (2021). Antibody response to 2-dose SARS-CoV-2 mRNA vaccine series in solid organ transplant recipients. JAMA.

[14] Herishanu Y, Avivi I, Aharon A, Shefer G, Levi S, Bronstein Y (2021). Efficacy of the BNT162b2 mRNA COVID-19 vaccine in patients with chronic lymphocytic leukemia. Blood.

[15] Goossens ME, Neven KY, Pannus P, Barbezange C, Thomas I, Van Gucht S (2021). Arch Public Health.

[16] Pannus P, Neven KY, De Craeye S, Heyndrickx L, Kerckhove SV, Georges D et al*.* Poor antibody response to BioNTech/Pfizer COVID-19 vaccination in SARS-CoV-2 naïve residents of nursing homes. medRxiv 2021; 2021.06.08.21258366.

[17] Mariën J, Ceulemans A, Michiels J, Heyndrickx L, Kerkhof K, Foque N (2021). Evaluating SARS-CoV-2 spike and nucleocapsid proteins as targets for antibody detection in severe and mild COVID-19 cases using a Luminex bead-based assay. J Virol Methods.

[18] Van Gassen S, Callebaut B, Van Helden MJ, Lambrecht BN, Demeester P, Dhaene T (2015). FlowSOM: using self-organizing maps for visualization and interpretation of cytometry data. Cytom J Int Soc Anal Cytol.

[19] Neumann J, Prezzemolo T, Vanderbeke L, Roca CP, Gerbaux M, Janssens S (2020). Increased IL-10-producing regulatory T cells are characteristic of severe cases of COVID-19. Clin Transl Immunol.

[20] Pasciuto E, Burton OT, Roca CP, Lagou V, Rajan WD, Theys T (2020). Microglia require CD4 T cells to complete the fetal-to-adult transition. Cell.

[21] Maneikis K, Šablauskas K, Ringelevičiūtė U, Vaitekėnaitė V, Čekauskienė R, Kryžauskaitė L (2021). Immunogenicity of the BNT162b2 COVID-19 mRNA vaccine and early clinical outcomes in patients with haematological malignancies in Lithuania: a national prospective cohort study. Lancet Haematol.

[22] Redjoul R, Le Bouter A, Beckerich F, Fourati S, Maury S (2021). Antibody response after second BNT162b2 dose in allogeneic HSCT recipients. Lancet Lond Engl.

[23] Castermans E, Hannon M, Dutrieux J, Humblet-Baron S, Seidel L, Cheynier R (2011). Thymic recovery after allogeneic hematopoietic cell transplantation with non-myeloablative conditioning is limited to patients younger than 60 years of age. Haematologica.

[24] Forcade E, Kim HT, Cutler C, Wang K, Alho AC, Nikiforow S (2016). Circulating T follicular helper cells with increased function during chronic graft-versus-host disease. Blood.

[25] Cordonnier C, Labopin M, Chesnel V, Ribaud P, De La Camara R, Martino R (2009). Randomized study of early versus late immunization with pneumococcal conjugate vaccine after allogeneic stem cell transplantation. Clin Infect Dis Off Publ Infect Dis Soc Am.

[26] Jaffe D, Papadopoulos EB, Young JW, O’reilly RJ, Prockop S, Kernan NA (2006). Immunogenicity of recombinant hepatitis B vaccine (rHBV) in recipients of unrelated or related allogeneic hematopoietic cell (HC) transplants. Blood.

[27] Conrad A, Perry M, Langlois M-E, Labussière-Wallet H, Barraco F, Ducastelle-Leprêtre S (2020). Efficacy and safety of revaccination against tetanus, diphtheria, haemophilus influenzae type b and hepatitis B virus in a prospective cohort of adult recipients of allogeneic hematopoietic stem cell transplantation. Biol Blood Marrow Transplant J Am Soc Blood Marrow Transplant.

[28] Mohty B, Bel M, Vukicevic M, Nagy M, Levrat E, Meier S (2011). Graft-versus-host disease is the major determinant of humoral responses to the AS03-adjuvanted influenza A/09/H1N1 vaccine in allogeneic hematopoietic stem cell transplant recipients. Haematologica.

[29] Perry C, Luttwak E, Balaban R, Shefer G, Morales MM, Aharon A (2021). Efficacy of the BNT162b2 mRNA COVID-19 vaccine in patients with B-cell non-Hodgkin lymphoma. Blood Adv.

[30] Apostolidis SA, Kakara M, Painter MM, Goel RR, Mathew D, Lenzi K et al. Altered cellular and humoral immune responses following SARS-CoV-2 mRNA vaccination in patients with multiple sclerosis on anti-CD20 therapy. medRxiv 2021; 2021.06.23.21259389. 10.1038/s41591-021-01507-2 PMC8604727 34522051

[31] Ram R, Hagin D, Kikozashvilli N, Freund T, Amit O, Bar-On Y (2021). Safety and immunogenicity of the BNT162b2 mRNA COVID-19 vaccine in patients after allogeneic HCT or CD19-based CART therapy-A single-center prospective cohort study. Transplant Cell Ther.

[32] Baron F, Storer B, Maris MB, Storek J, Piette F, Metcalf M (2006). Unrelated donor status and high donor age independently affect immunologic recovery after nonmyeloablative conditioning. Biol Blood Marrow Transplant J Am Soc Blood Marrow Transplant.

[33] Silva-Cayetano A, Foster WS, Innocentin S, Belij-Rammerstorfer S, Spencer AJ, Burton OT (2021). A booster dose enhances immunogenicity of the COVID-19 vaccine candidate ChAdOx1 nCoV-19 in aged mice. Med N Y N.

[34] Redjoul R, Le Bouter A, Parinet V, Fourati S, Maury S (2021). Antibody response after third BNT162b2 dose in recipients of allogeneic HSCT. Lancet Haematol.

