# Supplementary material for: Predictors of neutralizing antibody response to BNT162b2 vaccination in allogeneic hematopoietic stem cell transplant recipients
Source: J Hematol Oncol. 2021 Oct 24;14:174. doi: 10.1186/s13045-021-01190-3 (PMC8542409; doi:10.1186/s13045-021-01190-3)
Supplement: Supplementary file 1 — Additional file 1. Additional tables (n = 2) and figures (n = 7). [file 13045_2021_1190_MOESM1_ESM.doc]

**Supplemental table 1 :** flow cytometry panels.

**Panel 1:** myeloid and B-cell assessment

| **Antigen** | **Fluorochrome** | **Clone** | **Dilution** | **Manufacturer** | **Reference** | **Staining** |
| --- | --- | --- | --- | --- | --- | --- |
| Anti – IgD | FITC | IA6-2 | 1:33 | BD Biosciences | 562023 | EC |
| Anti – CD27 | BV650 | L128 | 1:50 | BD Biosciences | 563228 | EC |
| Anti – CD56 | APC-R700 | NCAM16.2 | 1:25 | BD Biosciences | 565140 | EC |
| Anti – CD16 | BUV395 | 3G8 | 1:25 | BD Biosciences | 563784 | EC |
| Anti – CD14 | APC-Cy7^TM^ | MOP9 | 1:25 | BD Biosciences | 557831 | EC |
| Anti – CD11c | PerCP^TM^-Cy5.5 | B-Ly6 | 1:33 | BD Biosciences | 565227 | EC |
| Anti – CD123 | PE | 6H6 | 1:25 | BD Biosciences | 566920 | EC |
| Anti – CD141 | BV711 | 1A4 | 1:25 | BD Biosciences | 563155 | EC |
| Anti HLA-DR | BV510 | G46-6 | 1:33 | BD Biosciences | 563083 | EC |
| Anti – CD19 | BV786 | SJ25C1 | 1:33 | BD Biosciences | 563325 | EC |
| Anti – CD3 | V450 | UCHT1 | 1:25 | BD Biosciences | 560365 | EC |
| Anti – CD33 | PE^TM^-Cy7 | P67.6 (CE/IVD) | 1:33 | BD Biosciences | 333952 | EC |
|  | eFluor 455UV |  |  | eBioscience^TM^ | 65-0868-14 | FVD |
| Anti – CD86 | APC | 2331 (FUN-1) | 1:33 | BD Biosciences | 555660 | IC |

**Panel 2:** T-cell subpopulation assessment

| **Antigen** | **Fluorochrome** | **Clone** | **Dilution** | **Manufacturer** | **Reference** | **Staining** |
| --- | --- | --- | --- | --- | --- | --- |
| Anti – CD4 | BV786 | SK3 | 1:50 | BD Biosciences | 563877 | EC |
| Anti – CD8a | BV510 | RPA-T8 | 1:50 | Biolegend® | 301048 | EC |
| Anti – CD45RA | AF®700 | HI100 | 1:33 | BD Biosciences | 560673 | EC |
| Anti – CCR4 | AF®647 | 1G1 | 1:25 | BD Biosciences | 557863 | EC |
| Anti – CCR2 | BV421^TM^ | K036C2 | 1:20 | Sony Biotechnology | 2386050 | EC |
| Anti – CXCR3 | PE | 1C6/CXCR3 | 1:25 | BD Biosciences | 560928 | EC |
| Anti – CXCR5 | AF488 | RF8B2 | 1:50 | BD Biosciences | 558112 | EC |
| Anti – CCR6 | PE-Cy7 | R6H1 | 1:33 | eBioscience^TM^ | 25-1969-42 | EC |
| Anti – CD25 | BUV395 | 2A3 | 1:25 | BD Biosciences | 564034 | EC |
| Anti PD-1 | BV650 | EH12.2H7 | 1:20 | Biolegend® | 329949 | EC |
| Anti – CD62L | APC-AF®780 | DREG56 | 1:50 | eBioscience^TM^ | 47-0629-42 | EC |
|  | eFluor 455UV |  |  | eBioscience^TM^ | 65-0868-14 | FVD |
| Anti – FoxP3 | PE-CF594 | 259D/C7 | 1:10 | BD Biosciences | 562421 | IC |

**Supplemental table 2**

**A) Factors associated with anti-RBD Ab response (i.e. anti-RBD titer >= 5 IU/mL) at day 49 after first vaccination (uni- and multivariate logistic regression).**

|  | Univariate | | Multivariate (AUC=0.978) | |
| --- | --- | --- | --- | --- |
| Variable | OR (IC95%) | p-value | OR (IC95%)* | p-value |
| Age at vaccination (yeas) | 0.90 (0.80-1.03) | 0.13 | 0.99 (0.88-1.12) | 0.95 |
| Days from transplantation to vaccination (log) | 1.11 (0.28-4.48) | 0.88 | 4.01 (0.32-50.5) | 0.28 |
| Moderate/severe chronic GVHD (Yes vs. No) | **0.014 (<0.001-0.33)** | **0.0080*** | **0.014 (<0.001-0.49)** | **0.018** |
| Rituximab <1 year before vaccination (Yes vs. No) | 0.92 (0.087-9.82) | 0.95 | 0.23 (0.010-5.18) | 0.36 |

* Firth correction.

**B) Factors associated with anti-RBD Ab titers at day 49 after first vaccination (uni- and multivariate linear regression).**

|  | Univariate | | Multivariate (R²=0.689) | |
| --- | --- | --- | --- | --- |
| Variable | Estimate (SE) | p-value | Estimate (SE) | p-value |
| Age at vaccination (yeas) | **-0.069 (0.030)** | **0.029** | -0.043 (0.021) | 0.053 |
| Days from transplantation to vaccination (log) | 0.99 (0.63) | 0.13 | 0.80 (0.40) | 0.052 |
| Moderate/severe chronic GVHD (Yes vs. No) | **-4.00 (0.78)** | **<0.0001** | **-3.87 (0.66)** | **<0.0001** |
| Rituximab <1 year before vaccination (Yes vs. No) | **-2.34 (1.06)** | **0.035** | **-2.79 (0.70)** | **0.0004** |

**C) Factors associated with detectable neutralizing antibodies against wild-type SARS-CoV-2 at day 49 after first vaccination (uni- and multivariate logistic regression).**

|  | Univariate | | Multivariate (AUC=0.947) | |
| --- | --- | --- | --- | --- |
| Variable | OR (IC95%) | p-value | OR (IC95%)* | p-value |
| Age at vaccination (yeas) | 0.95 (0.89-1.003) | 0.062 | 0.92 (0.84-1.01) | 0.092 |
| Days from transplantation to vaccination (log) | 3.33 (0.99-11.2) | 0.052 | **5.72 (1.02-32.2)** | **0.048** |
| Moderate/severe chronic GVHD (Yes vs. No) | **0.030 (0.001-0.66)** | **0.026*** | **0.023 (<0.001-0.62)** | **0.025** |
| Rituximab <1 year before vaccination (Yes vs. No) | 0.13 (0.014-1.19) | 0.071 | 0.045 (0.002-1.21) | 0.065 |

* Firth correction.

**D) Factors associated with titers of neutralizing antibodies against wild-type SARS-CoV-2 at day 49 after first vaccination (uni- and multivariate linear regression).**

|  | Univariate | | Multivariate (R²=0.617) | |
| --- | --- | --- | --- | --- |
| Variable | Estimate (SE) | p-value | Estimate (SE) | p-value |
| Age at vaccination (yeas) | **-0.033 (0.014)** | **0.020** | **-0.027 (0.011)** | **0.016** |
| Days from transplantation to vaccination (log) | **0.83 (0.26)** | **0.0033** | **0.77 (0.20)** | **0.0006** |
| Moderate/severe chronic GVHD (Yes vs. No) | **-1.31 (0.42)** | **0.0035** | **-1.18 (0.33)** | **0.0013** |
| Rituximab <1 year before vaccination (Yes vs. No) | -0.96 (0.49) | 0.061 | **-0.97 (0.36)** | **0.011** |

**Supplemental figure 1. Gating strategy.**

USM, unswitched memory B cells ; SM, class-switched memory B cells ; DN, double negative B cells ; TCM, central memory T cells ; TEM, effector memory T cells; Tfh, follicular helper CD4+ T cells.

**Supplemental figure 2. Adverse events.** Adverse events reported in the first 49 days after first vaccination (graded according to Common Terminology Criteria for Adverse Events (CTC) version 5.0).

**Supplemental figure 3. Anti-receptor-binding domain (RBD) SARS-CoV-2 IgG titers following BNT162b2 mRNA vaccination**. All allo-HCT patients received the first vaccine on day 0 and 39 of the 40 patients received the second vaccine on day 21. A) Evolution of Ab levels in patients with prior (n=2) or ongoing (n=1) SARS-CoV-2 infection (n=3). B) Evolution of Ab levels in naïve patients without moderate/severe chronic GVHD (n=28). C) Evolution of Ab levels in naïve patients with ongoing moderate/severe chronic GVHD (n=9).

**Supplemental figure 4.** **Suggestion for a higher B-cell frequency at baseline in SARS-CoV-2 naive allo-HCT patients with anti RBD-ab > 5 IU/mL at day 21**. PBMCs at baseline were isolated from allo-HCT patients with anti RBD-ab ≤ (n=18) or > (n=19) 5 IU/mL at day 21 and assessed through flow cytometry. Three samples (1 from non-responders and 2 from responders) creating staining artefacts in the t-SNE analysis were excluded for this specific analysis. (a) t-SNE representation of cell populations based on the expression of the phenotypic markers: CD3, CD19, CD14, CD16 and CD86. FlowSOM clusters were annotated based on (b) expression of phenotypic markers across each cluster. (c) Quantification of conventional B-cell subsets in patients with anti RBD-Ab ≤ ( “non responders”) or > ( “responders”) 5 IU/mL on day 21. The horizontal lines show the medians. (d) t-SNE representation of PBMC populations based on aforementioned phenotypic markers for each condition, responders versus non-responders. (e) Dendrogram showing the comparative similarity between individuals based on the Kolmogorov–Smirnov statistics calculated using the cross‐entropy distributions derived from t-SNE. This subfigure shows that the non-responders (in red) have a tendency to cluster together (the same for responders, in blue). (f) t-SNE representation of PBMC populations based on aforementioned phenotypic markers for each condition, namely responders versus non-responders, with each individual represented in a different color.

.

**Supplemental figure 5. Higher switched memory B-cell frequency at baseline in SARS-CoV-2 naive allo-HCT patients with anti-RBD Ab > 5 IU/mL at day 21**. PBMCs at baseline were isolated from allo-HCT patients with ant-RBD Ab ≤ (n=18) or > (n=19) 5 IU/mL at day 21 and assessed through flow cytometry. B cells were manually gated in FlowJo. (a) tSNE representation of B-cell cluster populations based on the expression of the phenotypic markers CD27, IgD, CD11c, CD86, HLA-DR. FlowSOM clusters were annotated based on (b) expression of phenotypic markers across each cluster. (c) Quantification (individual dots + median) of conventional B-cell subsets in patients with anti-RBD Ab ≤ ( “non responders”) or > ( “responders”) 5 IU/mL on day 21. (d) tSNE representation of PBMC populations based on aforementioned phenotypic markers for each condition, responders versus non responders. (e) Dendrogram showing the comparative similarity between individuals based on the Kolmogorov–Smirnov statistics calculated using the cross‐entropy distributions derived from tSNE. This subfigure shows that the non-responders (in red) have a tendency to cluster together (the same for responders, in blue). (f) tSNE representation of PBMC populations based on aforementioned phenotypic markers for each condition, namely responders versus non-responders, with each individual represented in a different color. Two samples containing few B cells (144 (red arrow in panel C) and 502 cells instead of 2700 cells in all other samples) were nevertheless included in the t-SNE analysis to avoid creating informative censuring.

**Supplemental figure 6. Higher plasmacytoid dendritic cell frequency at baseline in SARS-CoV-2 naive allo-HCT patients with anti RBD-ab > 5 IU/mL at day 21**. PBMCs at baseline were isolated from allo-HCT patients with anti RBD-ab ≤ (n=18) or > (n=19) 5 IU/mL at day 21 and assessed through flow cytometry. Myeloid cells were manually gated in FlowJo by excluding lymphocyte subsets (CD3, CD19 and CD56). Five samples (2 from non-responders and 3 from responders) creating staining artefacts in the t-SNE analysis were excluded for this specific analysis. (a) t-SNE representation of myeloid cell cluster populations based on the expression of the phenotypic markers: HLA-DR, Siglec-F, CD16, CD86, CD141, CD14, CD11c, and CD123. FlowSOM clusters were annotated based on (b) expression of phenotypic markers across each cluster. (c) Quantification of conventional B-cell subsets in patients with anti RBD-Ab ≤ ( “non responders”) or > ( “responders”) 5 IU/mL on day 21. The horizontal lines show the medians. (d) t-SNE representation of PBMC populations based on aforementioned phenotypic markers for each condition, responders versus non-responders. (e) Dendrogram showing the comparative similarity between individuals based on the Kolmogorov–Smirnov statistics calculated using the cross‐entropy distributions derived from t-SNE. This subfigure shows that the non-responders (in red) have a tendency to cluster together (the same for responders, in blue). (f) t-SNE representation of PBMC populations based on aforementioned phenotypic markers for each condition, namely responders versus non-responders, with each individual represented in a different color.

**Supplemental figure 7. Spearman r correlation matrix between day 49 anti-RBD Ab levels, day 49 50% neutralizing Ab titers of SARS-CoV-2 wild type, percentages of immune cells among absolute lymphocytes, and age in SARS-Cov2 naive allo-HCT recipients (n=37).** RBD, receptor-binding domain; Ab, antibody; SM B cells, class-switched memory B cells; UM B cells, class-unswitched memory B cells; TFH, T follicular helper cells.
